# Defaults at Work: A Field Experiment on the Effect of Nudges on Stand-Up Working

**DOI:** 10.3390/ijerph22070994

**Published:** 2025-06-24

**Authors:** Mathias Celis, Nicolas Dirix, Mona Bassleer, Wouter Duyck

**Affiliations:** 1 Department of Experimental Psychology, Faculty of Psychology and Educational Sciences, Ghent University, 9000 Ghent, Belgium; nicolas.dirix@ugent.be (N.D.); mona.bassleer@ugent.be (M.B.); wouter.duyck@ugent.be (W.D.); 2 NVAO, The Accreditation Organization of the Netherlands and Flanders, 2514 JG The Hague, The Netherlands

**Keywords:** occupational health, field experiment, default nudge, sedentary behavior, stand-up working, workplace health promotion, behavioral intervention, occupational physical activity, nudge transparency

## Abstract

Sedentary behavior at work is a major, and growing, public health concern. This field experiment investigates the effectiveness of behavioral nudges, specifically default settings on height-adjustable workstations (HAWS), in promoting stand-up working behavior. It also examines whether transparency and health coaching enhance these effects. The study was conducted in a Belgian governmental organization and included 149 employees across eight departments. Departments were randomly assigned to one of four conditions: a non-transparent default nudge (NTDN), a transparent default nudge (TDN), a classical health coaching intervention, or a hybrid intervention combining TDN and coaching. Over an eight-week intervention period, employee posture was recorded using fixed camera snapshots taken every 30 min. These data were used to calculate the stand-up ratio. The NTDN increased stand-up rates from 1.82% to 4.93%. The TDN more than doubled this effect, reaching 11.25%. The combination of TDN and coaching produced the highest increase, with stand-up rates rising to 18.80% (*d* = 9.85). Coaching alone showed no significant effect. Although behavior partially regressed after the interventions were removed, post-measurement stand-up ratios after a week remained higher than baseline. These findings suggest that transparent default nudges, especially when combined with low-threshold coaching, can meaningfully reduce sedentary behavior in everyday office environments.

## 1. Introduction

Recent studies on the effects of a sedentary lifestyle on occupational health risks paint a grim picture, with prolonged inactivity increasingly labeled as one of the major public health challenges of the 21st century [1,2,3]. Lee et al. [4] identify a sedentary lifestyle as the fourth largest risk factor for global deaths, associated with obesity, cardiovascular disease, diabetes, and (colon) cancer [5,6]. These studies show that we are globally moving at a rapid pace toward a lifestyle that increasingly invites sitting down for long periods of time.

Progressing urbanization, a rise in motorized transportation options, and the fact that people spend more and more of their free time on sedentary activities (such as watching TV and using computers) are partly behind these alarming global changes in physical activity (PA) [7,8]. It is therefore to be expected that this number will increase further under the influence of the rapid innovations in automation and computerization.

The Global Action Plan on Physical Activity (GAPA) by the World Health Organization (WHO) [9], aims to achieve a 15% relative decrease in global physical inactivity by 2030. However, recent WHO data indicates that 81% of adolescents and 27.5% of adults fail to meet the minimum activity requirements.

This global lack of physical activity has a large impact on population health and imposes a considerable economic burden [10]. Recent projections estimate that between 2020 and 2030, almost 500 million preventable cases of non-communicable diseases (NCDs) are expected to occur if current levels of physical inactivity persist [11]. This will result in healthcare costs of over USD 300 billion (INT$ 524 billion), amounting to approximately USD 27 billion (INT$ 48 billion) annually [12,13,14].

Most cases (75%) will arise in middle-income countries, with physical inactivity-related hypertension and depression accounting, respectively, for 47% and 43% of all new cases of NCDs [15,16]. High-income countries, although bearing the largest economic burden, will account for 70% of healthcare expenditure related to physical inactivity [9].

As shown by large-scale health surveys by Sciensano [17], this international trend is also manifesting itself in Belgium, where the current study takes place.

Belgium represents a relevant empirical context due to its notably high sedentary workforce and the lack of explicit national guidelines or workplace regulations aimed at reducing sedentary behavior, as reported by the WHO [18]. This policy gap presents both a challenge and an opportunity to examine the effectiveness of behavioral interventions like nudges in an under-regulated yet high-need environment.

The recent COVID-19 pandemic has reshaped how people work [19]. As a result of the sudden obligation to work remotely during the COVID-19 pandemic, the percentage of individuals (aged 18 years and older) sitting down for more than 8.5 h per day tripled (to 56.1%) compared to measurements before the COVID-19 crisis [17].

### 1.1. Exercise Is Not Enough

It is clear that engaging in PA is important to mitigate health risks and increase longevity [20]. However, most interventions are not aimed at reducing sedentary time, but at increasing moderate to vigorous physical activity (MVPA) [21]. Paradoxically, studies show that simply engaging in PA for just 30 min a day does not counteract the negative effects of prolonged sitting [22,23].

The key appears to lie in replacing sedentary time with light physical activity (LPA) like walking slowly, performing household chores, working while standing up, or by replacing it with MVPA (like taking the stairs, light jogging, swimming, walking the dog [24].

Adults who replace only thirty minutes of sitting time a day with LPA or MVPA, have a, respectively, 14% and 45% lower risk of dying from cardiovascular diseases and live on average three years longer than similarly inactive individuals [25]. Another large-scale study finds that adults who replace one hour of sedentary time with MVPA, reduce their mortality risk by up to 18% [26].

### 1.2. Sitting at Work

Occupational PA has decreased by over 30% globally, with 77% of blue-collar working hours now spent sedentary [27,28,29]. In this workplace, sitting accounts for over 60% of total sitting time and carries substantial economic costs of 4–6% of global GDP [30,31]. Despite the WHO calling for action, only 36% of countries have implemented workplace PA policies, with Belgium, where this study takes place, notably lacking such guidelines [9,18].

In response, many organizations have introduced workplace wellness initiatives aimed at reducing sitting time and promoting employee health [32,33,34]. These programs often include features such as on-site fitness options, mental health support, and the installation of height-adjustable workstations (HAWS), which allow employees to alternate between working while sitting or standing up.

Research has demonstrated that HAWS holds potential to replace employee sitting time with LPA and can thus significantly alleviate the detrimental health effects associated with prolonged sitting, as reported by [35]. This has been supported by findings that the use of HAWS at standing height leads to a rise in energy expenditure [36], improved concentration [37], heightened productivity [38], and a decrease in job related fatigue [39].

The implementation of HAWS can therefore be seen as an easy and cost-effective investment for organizations, as it offers employees an alternative to sitting down, with the ability to decrease the duration of sitting while at work, improving employee health, without the need to disengage from their workstations. However, research suggests that merely providing employees with HAWS is not sufficient to stimulate stand-up working, as they are primarily used at sitting height after the novelty factor has worn off [36]. These findings suggest that simply providing the facilities to work standing up is not sufficient to result in long-term behavioral change.

### 1.3. Interventions to Reduce Sedentary Behavior at Work

Shrestha et al.’s [40] review of thirty-four workplace interventions found that HAWS, alone or combined with information and counseling, reduced sitting time by 100 min per workday. In contrast, standalone informational interventions, including feedback, counseling, computer prompts, or mindfulness training, showed no significant effects. This pattern, confirmed by multiple meta-analyses, demonstrates that environmental modifications consistently outperform information-based approaches in reducing workplace sedentary behavior [41,42,43,44,45,46,47,48].

Traditional health behavior theories, including the Health Belief Model (HBM) [49], the Transtheoretical Model/Stages of Change (TTM) [50], Social Cognitive Theory (SCT) [51], the Social Ecological Model (SEM) [52], and the Theory of Reasoned Action/Theory of Planned Behavior (TRA/TPB) [53] assume fundamentally rational decision-making based on logical cost–benefit assessments.

This rationalistic view, however, fails to account for the powerful influence of contextual factors and a bounded rationality, which can subtly guide behavior in less conscious or non-rational ways [54]. Such influences and processes are underestimated in the current health behavior theories. These theories would therefore benefit from a more comprehensive approach that integrates both rational decision-making and non-rational effects, allowing for a more realistic and nuanced understanding of health behaviors, recognizing the complex interplay between conscious reasoning, environmental influences, and subconscious cues.

### 1.4. Nudging

Behavioral economics offers a cost-effective approach to addressing non-rational behavioral patterns through nudging, which involves shaping behavior without restricting choice options [55]. As early as the 1970s, the principles that underlie nudges have been established based on dual-processing models of human behavior [56].

While there are various dual-processing theories, they all recognize the existence of two separate processing modes, commonly metaphorically referred to as System 1 (automatic, intuitive, prone to biases) and System 2 (deliberate, conscious, effortful), which was popularized by [57]. Choice architects intervene in the physical environment to leverage both cognitive systems but primarily exploit System 1 biases to influence behavior where rational System 2 interventions typically fail. Of all System 1 biases, the tendency to maintain defaults and preset settings, a manifestation of cognitive laziness, and status quo bias, proves particularly powerful for behavior change.

By strategically altering what is presented as the default or pre-selected option, choice architects can guide behavior without requiring any active decision from individuals or restricting choice options. Default nudges are therefore the most powerful tool in the choice architect’s arsenal. For example, Johnson and Goldstein [58] demonstrated that switching organ donation from opt-in to opt-out dramatically increased participation rates, showing how minor changes in default settings can influence even fundamental personal decisions. Similarly, automatic enrollment in retirement savings programs with opt-out options increases participation by 50% [59,60,61]. Meta-analyses confirm default nudges have the highest effectiveness among all nudging interventions, with median effect sizes of 50% and d = 0.62 [62,63]. This effectiveness stems from eliminating the cognitive burden of decision-making while preserving freedom of choice [59,64,65].

Multiple reviews have examined workplace interventions promoting PA and reducing sedentary time [21,41,42,66,67,68,69,70,71]. Most nudging studies focused on increasing stair use through prompts like painted footsteps or social comparisons [42,48]. However, only eight nudging interventions specifically targeted workplace sedentary behavior, primarily using technology-based prompts, wearable vibration alerts, poster reminders, or walking meetings, with mostly inconclusive results [42]. Only three studies reported positive effects, all with significant limitations including minimal sample sizes ([72], *n* = 3; [73], *n* = 17). The application of default nudges to promote HAWS usage at standing height has been examined in only one field study to date: Venema et al. [74] investigated this intervention in a large governmental organization equipped with 110 HAWS among 836 desks. They placed all HAWS at standing height with signs requesting employees to leave desks in standing position, finding a 621.43% increase in standing behavior (from 1.82% to 13.13%) that partially persisted post-intervention (10.01% at two weeks, 7.78% at two months). However, this study had three key limitations: potential Hawthorne effects from visible researcher observation [75,76], lack of a hybrid intervention combining nudges with information despite evidence supporting such approaches [40], and transparent implementation that raises questions about transparency’s effect on nudge effectiveness.

### 1.5. Transparency in Nudging

Since its introduction as a policy instrument, the question of transparency (or the degree to which individuals are conscious of the existence and intended effect of a nudge) in nudge applications has been debated [77]. For example, some believe that nudge interventions manipulate people into choosing things they otherwise would not, as they often target nonconscious, automatic, System 1 processes [78]. Others argue that such interventions enhance autonomy to the extent that it facilitates the choice individuals would have made, given the choice [79]. Several systematic reviews and meta-analysis on nudge effectiveness and the underlying conditions that make nudges work, suggest that people’s choices are not as easily influenced by nudges as is generally believed [62,63,80].

Multiple studies provide evidence that disclosing the presence of a nudge [77,79,81], the underlying mechanism at work [81,82], or even its goal [81,83,84], does not significantly decrease its effectiveness, compared to situations where such information was not revealed, nor does it influence the decisionmaker’s experience of autonomy and choice satisfaction [65,85,86,87]. This has, however, not been studied in a real life, in-the-field work context.

Given the significant potential health benefits of reducing employee sedentary time using HAWS, it is relevant to investigate whether a default nudge can effectively stimulate employees to work more while standing up. We are also interested in the comparison of the effectiveness of a default nudge with more classical intervention methods such as a health coaching intervention, both as standalone interventions as well as combined with a default nudge intervention.

Prior study results by Zimmermann and Renaud [87] suggest that a combination of a nudge and information provision, referred to as a hybrid nudge, is equally or sometimes even more effective in some decision-making scenarios in influencing decision outcomes. Lastly, there is still a large gap in the research literature on the effectiveness of nudge transparency in a ‘real life’ workplace setting. We aim to specifically study the influence of nudge transparency on default nudge effectiveness when placing HAWS at standing height.

### 1.6. Current Study

The objective of the current study is to examine how the different behavioral interventions (non-transparent default nudges (NTDN), transparent default nudges (TDN), coaching, and a hybrid of both) affect stand-up working behavior in a real-world office setting. Additionally, the study aims to examine the effect of transparency (or lack thereof) on the effectiveness of a default nudge in a work setting. Furthermore, this study aims to investigate the effectiveness of a hybrid intervention that consists of a coaching intervention in combination with TDN.

Based on prior findings regarding the overall effectiveness of default nudges, we expect that implementing a default nudge will reduce sedentary behavior at work [61,74,84,88]. In accordance with findings by Paunov et al. [89] and Steffel et al. [84], we expect that nudge transparency will positively influence its effectiveness. In line with findings by Shrestha et al. [40] we believe that a hybrid intervention will yield better results than either standalone intervention, as a default nudge exploits the tendency of people to stick with the preset option due to inertia, while information provision makes the desirable option more salient and noticeable. This way, the hybrid form may appeal to both automatic (System 1) and reflective (System 2) decision-making systems and increase the likelihood of going with the desirable option [90]. We assume that all the interventions will result in an increase in the utilization of HAWS at standing height (expressed as an increase in stand-up ratio) in comparison to a control condition, as well as compared to their baseline and post-intervention measurements.

## 2. Methods

### 2.1. Participants

This quasi-experimental field study was conducted at a medium-sized Belgian government organization. The workplace consisted of an open-plan office with flexible seating, where employees can choose any available height-adjustable workstation (HAWS) on their floor upon arrival. A noteworthy aspect of the organizational layout is that departments were physically located on separate floors or in enclosed zones of the building, such that employees from different departments had no direct line of sight or structural visual exposure to other groups. The sample consisted of eight distinct departments, comprising a total of 149 employees. All participants had predominantly sedentary job duties. Observations at baseline revealed that the default height of the HAWS in all eight departments was set at sitting height. The participants were aged between 20 and 65, with a mean age of 41.92 years (SD = 11.09) and a gender distribution of 25.88% male and 74.12% female, which was representative of the organization’s employee population. Participants were randomly assigned to a condition on department level by tally, using simple randomization. Each department was randomly allocated to a condition by drawing lots, ensuring an unbiased distribution across experimental groups while preserving the department as the unit of assignment. Departments were selected based on the criterion that all employees performed predominantly sedentary, office-based tasks. All employees within these departments were included to ensure ecological validity and minimize selection bias.

### 2.2. Design and Procedure

The experiment ran for eight weeks: four weeks of baseline sit/stand behavior, two weeks of intervention and one week of follow-up measurement. Using eight camera devices, spread across the departments, 3840 h of data and 91,604 datapoints were collected. All interventions were implemented directly by the lead researcher. The default nudge intervention, raising all HAWS to standing height, was applied manually each evening after 9:00 PM, once the workplace was empty, to ensure participants would encounter the altered setting at the start of their workday without prior notice. The coaching interventions were also delivered by the lead researcher, who was formally trained in the intervention material. The intervention study followed a two-phased sequential design.

#### 2.2.1. Phase 1: Non-Transparent

The first phase of the study aimed to investigate the effect of a default nudge in the absence of nudge transparency to the participants. Because the second phase implies nudge awareness, this non-transparent phase was necessarily applied first for all participants. Eight departments (*n* = 149) were randomly assigned to either the control group (*n* = 66), who received no intervention or to the default nudge group (*n* = 83).

The HAWS of the departments that got allocated to the NTDN group were changed from sitting height to standing height before the start of every workday, during a one-week intervention. No disclosure or signaling on why desks were put at standing height was provided in this stage of the experiment. The week after concluding the one-week non-transparent intervention, no intervention took place. At the end of this week, participants in the default group received an email that provided context and transparency on the nudge aim and mechanism.

#### 2.2.2. Phase 2: Transparent

For the second phase of the study, four conditions were created. Participants that were allocated to the control group in the first phase were now either assigned to a control group or a health coaching intervention. The former received no intervention; the latter received an activating, informational group health coaching session on the risks of a sedentary lifestyle. To maximize the ecological validity of the health coaching intervention, we used the materials provided by a Belgian external service for prevention and safety at work. These materials are an integral part of the educational health & safety offering, called “Move@work”. Due to intellectual property restrictions, the training content used in this intervention is not publicly available for reproduction or publication.

Additionally, participants in this condition received a set of low-barrier movement tips and tricks with the goal of stimulating using their HAWS in a standing position. As part of the coaching session, participants were also asked to formulate a movement plan, for which they had to identify when, under what concrete circumstances, with which colleague, for how long, … they would use their HAWS in a standing position.

Participants that were allocated to the default condition in phase 1 were assigned to either (1) the same default nudge or (2) default nudge + health coaching (~hybrid intervention). For phase 2, every HAWS in the groups that included a TDN also received a sign taped to their HAWS that kindly asked the employee to leave the HAWS at standing height when leaving their desk/office. The sign further depicted two images to give an indication of the appropriate ergonomic height to set the desk in both a sitting and standing height.

The effectiveness of the same default nudge (*n* = 41), an activating workshop (*n* = 28), and a combination of both (*n* = 41) in comparison to the control group (38) were examined.

To test our hypotheses, we compared each intervention condition with a control group, as well as with each other. Specifically, the following condition pairs were analyzed: NTDN vs. Coaching, NTDN vs. TDN, TDN vs. Coaching, TDN vs. Hybrid, and Hybrid vs. Coaching. These comparisons were conducted across three measurement periods: baseline, intervention, and post-intervention. In addition, within-condition comparisons were made between pre-, during-, and post-intervention measurements to assess change over time within each group.

## 3. Measures

### 3.1. Stand-Up Ratio

The standing behavior of the participants was measured using eight ceiling-mounted camera devices, strategically placed so that they covered each department’s work area. This placement allowed for passive and unobtrusive observation during working hours. To assess the impact of each intervention, the number of desks in use was recorded every thirty minutes during working hours, for eight weeks. The “stand-up ratio” was calculated by dividing the number of standing employees by the number of total employees present, multiplied by 100. This ratio was then averaged per intervention, over all measurements. This proportion was calculated for each time point and subsequently averaged across the relevant measurement period. This operationalization follows procedures established by Venema et al. [74].

### 3.2. Analyses

The data collected in this study is analyzed using descriptive statistics, a 2 × 4 two-way ANOVA to investigate the effect of the measurement moment and the intervention type on the stand-up ratio (NTDN, TDN, coaching intervention, and the combined intervention of a TDN with coaching intervention) on the stand-up ratio. We use Bonferroni-Adjusted Pairwise Comparisons to further examine the two-way interaction between and within the different interventions. The central dependent variable for all conditions is the stand-up ratio (the proportion of standing employees, divided by the total present employees per department, multiplied by 100).

Due to the open-plan and flexible-seating nature of the workplace, it was not possible to link posture observations to individual demographic data such as age or gender. These variables were recorded only at the aggregate sample level and used for descriptive purposes. Individual-level subgroup analysis was not part of the study design, as the behavioral outcome data were not individually tracked.

### 3.3. Ethical Considerations

Written ethical approval was obtained by the company’s labor union and the managerial board. Prior to conducting the study, the true nature and research objectives were not disclosed to participants in order not to compromise the influence of intervention transparency on stand-up behavior. Participants were informed that an exploratory study involving cameras would be conducted to study workplace behavior. This information was conveyed to participants through an informational letter, provided in Dutch, and a group information session. The letter informed participants of the observation of their team’s work conditions and the placement of cameras for a number of weeks. The letter stated that all employees would be made unrecognizable in the footage and that only the researcher would have access to the footage, which would be destroyed after the necessary observations were made.

As during the first four weeks of the study, only baseline measurements were gathered, no actual interventions were administered nor noticeable, which minimizes awareness and Hawthorn effects. Employees were asked to work as they would normally. Additionally, signalization was placed in all areas where cameras were present, and employees were invited to contact a designated employee representative with any questions or concerns. The study was conducted in accordance with GDPR law, with all necessary measures implemented to fulfill ethical standards for conducting scientific research. Upon completion of the experiment, participants were informed of the true nature and research objectives of the study.

## 4. Results

A two-way ANOVA was used to investigate the effects of the measurement moment and the intervention type on the stand-up ratio. An overview of the descriptive statistics (means and standard deviations) for each intervention group across the three measurement moments can be found in Table S1 of the Supplementary Materials (SM).

First, a significant main effect of the measurement moment (*F*(2, 135) = 593.79, *p* < 0.001, *d* = 5.93) is found. The stand-up ratio is significantly higher during intervention (*M* = 7.90, *SD* = 6.60) than both during baseline (*M* = 1.86, *SD* = 0.66) ($\left|∆M\right|=6.04$, *p* < 0.001, *d* = 1.29) and post-measurement (*M* = 4.74, *SD* = 3.39) ($\left|∆M\right|=3.16$, *p* < 0.001, *d* = 0.6). In addition, the stand-up ratio during post-measurement is significantly higher than during baseline ($\left|∆M\right|=2.88$, *p* < 0.001, *d* = 1.18).

Second, the main effect of the intervention type is also significant (*F*(4, 135) = 381.24, *p* < 0.001, *d* = 6.74). The highest stand-up ratio occurs for the combination TDN-coaching group (*M* = 6.14, *SD* = 6.74), followed by the TDN group (*M* = 4.00, *SD* = 3.65) and the NTDN group (*M* = 2.58, *SD* = 1.29). The lowest stand-up ratios are found for the coaching (*M* = 2.28, *SD* = 0.79) and control group (*M* = 1.74, *SD* = 0.53). The difference in stand-up ratio is only non-significant between these latter two groups.

**Figure 1:**
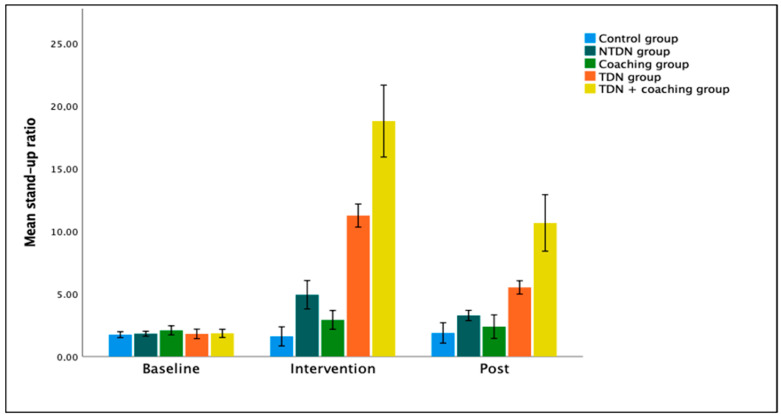
Two-way interaction measurement moment and intervention type. Note. NTDN = Non-transparent default nudge, TDN = Transparent default nudge; Baseline = 4 weeks of measurement; Intervention = one week of measurement; Post = one week of measurement (the week after removing the intervention. Stand-up ratio means during baseline, intervention, and post-measurement for all intervention types. The error bars represent the 95% confidence intervals of the means. NTDN = non-transparent default nudge, TDN = transparent default nudge. The post-measurement took place the week after removing the intervention.

The crucial interaction of measurement moment and intervention was significant, (*F*(8, 135) = 169.46, *p* < 0.001, *d* = 6.32). At baseline, there was no main effect of intervention (*F*(4, 99) = 0.82, *p* = 0.52). After the intervention, there was a main effect of intervention type (*F*(4, 24) = 70.32, *p* < 0.05). Below, we discuss Bonferroni-Adjusted Pairwise Comparisons to further examine this interaction effect. This two-way interaction is visualized in Figure 1. During baseline, there were no significant differences between any intervention types (mean difference range [0.02; 0.35]).

### 4.1. Non-Transparent Default Nudge (NTDN) and Coaching

Comparisons of the stand-up ratios of the *NTDN group* and the *control group* reveal that the stand-up ratio is significantly higher for the NTDN group during the intervention ($\left|∆M\right|=3.32$, *p* < 0.001, *d* = 4.26). The difference in stand-up ratio is non-significant during post-measurement ($\left|∆M\right|=0.08$, *p* > 0.05).

Further, the stand-up ratios between the *coaching and control groups* differ non-significantly during the intervention ($\left|∆M\right|=1.32$, *p* = 0.118) as well as during post-measurement ($\left|∆M\right|=0.50$, *p* = 1.000).

For the comparison of the stand-up ratios of the *NTDN and coaching groups*, a significant higher stand-up ratio is found for the NTDN group during the intervention ($\left|∆M\right|=2.01$ *p* = 0.002, *d* = 2.61). The difference in stand-up ratio is non-significant during post-measurement ($\left|∆M\right|=0.89$, *p* = 0.857).

### 4.2. Non-Transparent Default Nudge and Transparent Default Nudge

The stand-up ratio of the *TDN group* compared with the *control group* is significantly higher for the TDN group during the intervention ($\left|∆M\right|=9.65$, *p* < 0.001, *d* = 14.12). A similar finding occurs during post-measurement ($\left|∆M\right|=3.63$, *p* < 0.001, *d* = 6.52).

When comparing the stand-up ratios of the *NTDN and TDN groups*, the stand-up ratio during the intervention was significantly lower for the NTDN group compared with the TDN group ($\left|∆M\right|=6.33$, *p* < 0.001, *d* = 7.62). A similar pattern occurs during post-measurement ($\left|∆M\right|=2.24$, *p* < 0.001, *d* = 5.84).

### 4.3. Coaching, Transparent Default Nudge, and Combination TDN-Coaching

A significant higher stand-up ratio of the *combination TDN-coaching* compared with the *control group* is found during the intervention ($\left|∆M\right|=17.19$, *p* < 0.001, *d* = 10.16). A similar finding also appears during post-measurement ($\left|∆M\right|=8.78$, *p* < 0.001, *d* = 6.45).

When comparing the *coaching and TDN groups*, the stand-up ratio of the TDN group is significantly higher during the intervention ($\left|∆M\right|=8.33$, *p* < 0.001, *d* = 12.37) and during post-measurement ($\left|∆M\right|=3.13$, *p* < 0.001, *d* = 5.07).

When making the comparison between the *TDN and combination TDN-coaching groups*, the TDN group shows a significant higher stand-up ratio during the intervention ($\left|∆M\right|=7.54$, *p* < 0.001, *d* = 4.4) and during post-measurement ($\left|∆M\right|=5.15$, *p* < 0.001, *d* = 3.91).

As a final comparison, we studied the difference in stand-up ratio between the *coaching and combination TDN-coaching groups*. The stand-up ratio of the combination TDN-coaching group is significantly higher, both during the intervention ($\left|∆M\right|=15.88$, *p* < 0.001, *d* = 9.41) and during the post-measurement ($\left|∆M\right|=8.28$, *p* < 0.001, *d* = 5.96).

### 4.4. Within Intervention Types

Within the *control group*, no significant difference in stand-up ratio is found during the intervention, pre- and post-measurement (mean difference range $\left[0.14;0.28\right]$, all *p*’s > 0.05).

Within the *NTDN, TDN, and combination TDN-coaching groups*, the stand-up ratio during the intervention is significantly higher than during baseline (mean difference range $\left[3.11;16.95\right]$, all *p*’s < 0.001, *d* range $\left[4.39;9.94\right]$) and post-measurement (mean difference range $\left[1.65;8.13\right]$, all *p*’s < 0.005, *d* range $\left[2.43;9.48\right]$). Within the *coaching group*, the differences in stand-up ratio between the different measurement moments are non-significant (mean difference range $\left[0.29;0.83\right]$, all *p*’s > 0.050).

## 5. Discussion

Prolonged sedentary behavior remains a major public health concern, with workplace sitting identified as a key contributor [3,20,22]. This study assessed the effectiveness of three behavioral interventions: non-transparent default nudges (NTDN), health coaching, and a hybrid approach, all aimed at increasing the stand-up ratio during work, using height-adjustable workstations (HAWS).

### 5.1. Non-Transparent Default Nudge (NTDN) and Coaching

The non-transparent default nudge significantly increased HAWS usage at standing height by 170.88% (from a pre-measurement stand-up ratio of 1.82% to 4.93% during the intervention), aligning with prior meta-analytic findings that identify default nudges as among the most effective behavioral interventions [62,82].

In contrast, the standalone coaching intervention yielded no significant effect, echoing evidence that information alone often fails to overcome contextual and behavioral barriers to change [40,91].

### 5.2. Non-Transparent Default Nudge and Transparent Default Nudge

The transparent default nudge (TDN) increased standing behavior by 521.55% (from 1.81% to 11.25%), substantially outperforming the NTDN’s 170.88% increase. This finding directly contradicts Bovens’ [92] assertion that nudges work best “in the dark” and instead supports emerging evidence that transparency enhances nudge effectiveness [77,81,86].

The superior performance of TDN can be explained through dual-process theory [57]. While both nudges engage System 1 (automatic) processing by altering the default, transparency additionally activates System 2 (reflective) processing, allowing individuals to consciously align their behavior with the intervention’s health goals. This conscious engagement may strengthen commitment and reduce psychological reactance [93].

Critically, the NTDN triggered substantial reactance, resulting in three formal workplace bullying complaints with the *external service for prevention and protection at work*. Employees developed conspiracy theories about the daily desk adjustments (e.g., “*This is a passive aggressive strike action of the cleaning team, because employees fail to empty their trashcans in time*” or “*We think this is the work of the marketing department*”), demonstrating how a lack of transparency can threaten perceived autonomy and trigger resistance [94]. This reactance finding aligns with Steindl et al. [93], who showed that transparency about intervention goals and processes reduces workplace resistance.

Our results closely parallel Venema et al. [74], who reported a 621.43% increase using a similar transparent intervention. The slight difference (521.55% vs. 621.43%) may reflect our less intrusive implementation, adjusting desks only once daily rather than multiple times, suggesting a dose–response relationship between nudge frequency and effectiveness.

### 5.3. Hybrid Intervention (Coaching + Transparent Default Nudge)

The hybrid intervention produced the largest effect, increasing standing behavior by 921.74% (from 1.84% to 18.80%), despite coaching alone showing no significant effect. This synergistic outcome supports the Capability, Opportunity, Motivation Behavior (COM-B) model [95], where behavior change requires all three components: coaching provides capability (knowledge), the default nudge creates opportunity (environmental facilitator), and their combination enhances motivation through social norm formation.

The default nudge appears to eliminate key behavioral barriers identified in the literature. By making standing the default for multiple employees simultaneously, it addresses pluralistic ignorance, the false belief that one’s peers prefer sitting [96]. We believe this lowers the feeling of being perceived by colleagues as ‘the only hipster that works while standing up’ and the awkwardness of feeling that you are in the spotlight. When employees observe colleagues standing, descriptive norms shift, creating a self-reinforcing cycle of behavior change [97]. This mechanism explains why coaching alone fails: knowledge without environmental support cannot overcome social barriers [98,99].

The exceptional effect size (921.74%) partly reflects the low baseline standing ratio (1.84%), demonstrating ceiling effects in reverse—behaviors with minimal baseline prevalence show proportionally larger improvements. This finding aligns with Shrestha et al.’s [40] meta-analysis showing HAWS interventions reduce sitting by 100 min daily when combined with informational support. Our results suggest that targeting extremely low-frequency behaviors may yield disproportionate health gains, particularly when interventions address multiple behavioral determinants simultaneously.

### 5.4. Long Term Effect

Nudge extinction, the gradual fading of nudge effects post-removal, represents a fundamental challenge to behavioral interventions [100,101]. One-week post-intervention, standing ratios declined across all nudge conditions: NTDN dropped 33.67% (from 4.93% to 3.27%), TDN dropped 51% (from 11.25% to 5.51%), and the hybrid intervention dropped 43.30% (from 18.80% to 10.66%). Notably, coaching showed no extinction, as it produced no initial effect.

Despite these declines, all nudge interventions maintained standing ratios significantly above baseline, suggesting partial habit formation. The pattern aligns with habit theory [102], where behaviors practiced in stable contexts can persist even when initial prompts are removed. The TDN’s steeper decline (51%) compared to NTDN (33.67%) may reflect greater reliance on conscious motivation, which wanes faster than automatic responses [103].

Our extinction rates exceed those reported by Venema et al. [74]: a 23.08% decline after two weeks and 40.44% after two months. This difference likely stems from implementation intensity; Venema et al. adjusted desks multiple times daily versus our once-daily approach, suggesting a dose–response relationship between nudge frequency and habit strength. Their longer follow-up revealed continued decay, highlighting the need for sustained environmental support or policy implementation (e.g., a “high desk policy” analogous to clean desk policies).

In contrast to the aforementioned study, our study only looked at the effects of the interventions one week after intervention removal; we can therefore not make claims on the long-term effects of the interventions we tested.

While the observed decline in stand-up behavior one week after the intervention suggests partial nudge extinction, it remains unclear how these effects evolve over a longer timeframe. Unfortunately, we were unable to collect additional post-intervention data to explore this further. Given the practical, real-world nature of the study, and the intrusiveness of sustained camera monitoring in the work environment, our observation window was capped at eight weeks. The study permit, granted by the company’s labor union and management board, explicitly limited the duration of data collection and was not extended for follow-up research. Future studies should aim to incorporate longer follow-up periods under broader consent frameworks, in order to more fully capture the temporal dynamics of behavioral change.

These findings parallel digital health intervention patterns, where 45% of users abandon fitness apps within three months due to novelty decay [104]. Both contexts suggest that maintaining behavior change requires either continuous environmental modification or transition to intrinsic motivation—neither of which single-shot interventions typically achieve.

### 5.5. Implications for Practice and Policy

The results of this study have important implications for theory, practice, and policy. By utilizing nudges and transparent communication in the workplace, this study demonstrates that standing behavior can be promoted while sedentary behavior can be reduced. It also highlights the crucial role that transparency plays in the success of these workplace behavioral interventions. Without transparency, reactance may occur, which can hinder the success of the interventions. These findings align with previous research indicating that transparency about the intervention’s goals and processes can reduce reactance.

In addition, this study emphasizes the importance of considering contextual factors and behavioral barriers when designing interventions to promote health behavior changes. While educational interventions are useful in providing employees with knowledge and strategies to encourage standing behavior, they may not be enough to result in behavioral change on their own. To effectively promote standing behavior at work, interventions that address both informational and behavioral barriers are likely necessary.

From a practical standpoint, these findings suggest that default nudges and transparent communication can be effective tools for workplace health promotion programs aimed at encouraging standing behavior and reducing sedentary behavior. Employers can use these strategies to promote the use of HAWS at standing height, rather than sitting height. Combining an educational intervention with a default nudge may also be an effective approach to promoting standing behavior at work.

Furthermore, the findings of this study also hold significant practical relevance, as the insights gained can be readily applied in diverse professional contexts. The behavioral interventions we present in this study have proven to be effective and require minimal maintenance, making them ideal for promoting healthy behaviors in the workplace and can thus positively contribute towards attaining the GAPA goal to achieve a 15% relative reduction in the global prevalence of physical inactivity by 2030 [105]. Our study allows for evaluating the effectiveness of different interventions within the same study context, providing a more granular insight into what works and what does not to get employees to move more at work.

In terms of policy implications, this study highlights the importance of workplace health and safety regulations that address sedentary behavior. Policymakers should consider incorporating default nudges and transparent communication strategies in workplace health promotion policies aimed at reducing sedentary behavior. Employers should also be encouraged to provide their employees with access to height-adjustable standing desks and to promote standing behavior at work.

### 5.6. Limitations and Guidelines for Future Research

First, this study was conducted in a single workplace, which may limit the generalizability of the results to other settings. Other workplaces may have different physical layouts, cultures, or work demands that affect the effectiveness of the interventions. Future research could aim to replicate the study in other workplaces to assess the generalizability of the findings.

A possible second limitation of the study is the relatively short duration of the intervention, which raises questions about the persistence of the observed effects on standing behavior. Previous research by Pronk et al. [36], indicates that providing employees with height-adjustable workstations (HAWS) may not be sufficient to maintain stand-up working behavior in the long term, as the novelty wears off and employees return to their previous sitting habits. Thus, it is possible that the effects of the current interventions may diminish once the novelty has worn off after a few weeks. Future research could investigate the durability of the interventions’ effects over longer periods.

Third, a potential limitation concerns observation bias. Although the study relied on minimally intrusive, ceiling-mounted devices to passively monitor standing behavior, participants were aware that a study was taking place, which could introduce observer effects. However, several study design elements were introduced to reduce this potential concern. For example, a four-week baseline period prior to any intervention allowed participants to habituate to the presence of the equipment. Also, the notable expressions of reactance during the non-transparent default intervention suggest that behavioral changes were more likely linked to the content and nature of the interventions than to mere observation. Nonetheless, future research could include additional blinding measures or a non-intervention control group to further isolate the impact of observational awareness.

Fortunately, Venema et al. [74] investigated the longer-term effects after removing the default nudge intervention. Their findings suggest that the effect on stand-up working behavior persisted for at least two months after the intervention was removed. This provides some assurance that the current interventions may have longer-term effects beyond the initial novelty period. However, additional research is needed to assess the sustainability of these effects over even longer periods. Future research should examine the effectiveness of these interventions over a longer period and in different workplace settings and whether they would sustain when implemented as a ‘high desk policy’, where employees are asked to always leave their desks at standing height, by company policy. We find, to a certain degree an extinction effect after removing the nudge intervention. Future studies can specifically aim at designing interventions that can sustain behavior change over a long timeframe, even after the nudge has been removed.

Another important limitation of this study is the potential for contamination between intervention and control groups, particularly during the implementation of the transparent default nudge (TDN). Despite random assignment at the department level and physical separation across different floors, the open-plan office environment and informal communication among employees may have led to cross-condition spillover. Employees in the control group may have become aware of the interventions through colleagues who were exposed to the TDN, potentially diluting the observed differences between conditions. This risk of contamination is inherent to real-world field experiments, especially in organizational settings where physical and informational boundaries are porous. Future studies should consider staggered roll-outs or implement stricter spatial or communication barriers to further mitigate these effects.

The flexible, non-assigned seating in the open-plan office and the aggregate nature of behavioral observations further prevented linking standing behavior to specific individuals. As such, subgroup analyses were not feasible. Future studies using individual-level tracking or stratified sampling designs, where practically and ethically feasible, could investigate demographic moderators such as gender, age, marital status or job role.

Lastly, this study did not examine the cost-effectiveness of the interventions, which may be an important consideration for employers and policymakers. It is possible that some interventions, such as providing standing desks, may be more expensive than others, such as computer prompts. Future research could explore the costs and benefits of different interventions to help employers and policymakers make informed decisions.

Employers have the opportunity to evaluate the prevailing ‘default’ work environment presented to their employees and modify elements that promote sedentary behavior with more active alternatives. Such interventions have the potential to enhance health outcomes, productivity, and reduce healthcare costs associated with prolonged sedentary behavior. To foster a culture of movement and reduce sedentary practices at work, several future guidelines can be recommended. One such approach involves encouraging standing or walking meetings as opposed to sedentary conference room gatherings. This not only promotes movement but also enhances meeting efficiency. Another approach involves incorporating active breaks, such as stretching, walking, or exercise routines, into the workday. Providing access to on-site fitness facilities or group fitness classes during lunch breaks or after work hours can further reinforce this initiative. Additionally, adjustable workstations can be provided to enable employees to alternate between sitting and standing positions, thereby mitigating the negative effects of prolonged sitting.

## 6. Conclusions

This study aimed to evaluate the effectiveness of behavioral interventions, specifically default nudges and health coaching, in promoting standing behavior among sedentary office workers. The findings confirm that default nudge interventions can be very effective tools to change hard-wired movement patterns at work. When being transparent on the aim and origin of the intervention, the effect rises significantly. Coaching interventions alone had no measurable effect, but when paired with a transparent default nudge, it further amplified behavioral change. These results underscore the importance of altering environmental defaults and communicating intervention intent clearly to overcome habitual sedentary behavior. Transparent default nudging, with or without supplemental coaching, emerges as the most effective and scalable strategy for encouraging active work routines.

## Data Availability

The data presented in this study are available on request from the corresponding author. Restricted access to the data was part of the agreement with the host institution to safeguard participant confidentiality and organizational privacy.

## Supplementary Materials

The following supporting information can be downloaded at: https://www.mdpi.com/article/10.3390/ijerph22070994/s1, Table S1. Descriptive Statistics.
