# Supplementary material for: Defaults at Work: A Field Experiment on the Effect of Nudges on Stand-Up Working
Source: Int J Environ Res Public Health. 2025 Jun 24;22(7):994. doi: 10.3390/ijerph22070994 (PMC12294546; doi:10.3390/ijerph22070994)
Supplement: Supplementary file 1 [file ijerph-22-00994-s001.zip › ijerph-3665694-supplementary.pdf]

SUPPLEMENTARY INFORMATION FOR

DEFAULTS AT WORK.

A FIELD EXPERIMENT ON THE EFFECT OF A DEFAULT NUDGE ON STAND-UP WORKING

Tab s1

Descriptive Statistics

Intervention type	Measurement	*M*	*SD*	*n*	*Observations*
Control group	Baseline	1.74	.50	20	15,151
	Intervention	1.61	.62	5	3,723
	Post	1.88	.66	5	3,811
	Total	1.74	.53	30	22,685
Non-transparent default nudge	Baseline	1.82	.42	20	19,633
	Intervention	4.93	.91	5	4,907
	Post	3.27	.33	5	4,888
	Total	2.58	1.29	30	29,428
Transparent default nudge	Baseline	1.81	.81	20	9,922
	Intervention	11.25	.74	5	2,479
	Post	5.51	.43	5	2,453
	Total	4.00	3.65	30	14,854
Coaching group	Baseline	2.09	.77	20	6,711
	Intervention	2.92	.60	5	1,680
	Post	2.38	.76	5	1,684
	Total	2.28	.79	30	10,075
Nudge + coaching group	Baseline	1.84	.70	20	9,711
	Intervention	18.80	2.31	5	2,459
	Post	10.66	1.81	5	2,392
	Total	6.14	6.74	30	14,562
Total	Baseline	1.86	.66	100	61,128
	Intervention	7.90	6.60	25	15,248
	Post	4.74	3.39	25	15,288
	Total	3.35	3.80	150	91,604

Note. The *n* in this table represents the amount of observation days of which we calculated the mean stand-up ratios.
